# Anxiety Reduction and Improved Concentration in Schoolchildren through Wingwave^®^ Coaching

**DOI:** 10.3390/children8121102

**Published:** 2021-11-30

**Authors:** Frank P. G. Weiland, Marco Rathschlag, Stefanie Klatt

**Affiliations:** Institute of Exercise Training and Sport Informatics, German Sport University Cologne, 50933 Cologne, Germany; fpgweiland@gmail.com (F.P.G.W.); m.rathschlag@dshs-koeln.de (M.R.)

**Keywords:** wingwave^®^ coaching, schoolchildren coaching, anxiety reduction, short-term coaching, concentration enhancement

## Abstract

(1) Background: For nearly 20 years, the wingwave^®^ method, which combines elements of eye movement desensitization and reprocessing (EMDR) and a muscular strength test, has been used to reduce anxiety and improve relaxation in subjects. Past studies have scientifically evaluated this method in various contexts and have found it to be effective. In this study, we investigated the effects of short-term wingwave^®^ coaching on specific anxiety parameters regarding school, concentration ability, and subjective feelings towards two self-chosen themes in schoolchildren. (2) Methods: A group of 53 schoolchildren aged 11 to 12 years were randomly divided between an experimental and a control group. The experimental group received an intervention of three wingwave^®^ coaching sessions (one hour each). In these sessions, past and present negative feelings towards school as well as psychological resources to face future tasks in school were focused on and utilized. (3) Results: The results showed that the overall text anxiety, manifested anxiety, and dislike of school decreased significantly in the experimental group after the three coaching sessions compared to the control group. Furthermore, both concentration ability and the subjective feeling towards self-chosen subjects improved significantly in the experimental group compared to the control group. (4) Conclusions: Our results indicate that the wingwave^®^ method is an appropriate and effective instrument to reduce school anxiety and to improve concentration performance in schoolchildren—at least in the short and medium term.

## 1. Introduction

It is critical to recognize emotions in order to understand human beings and their abilities and tendencies to cope with life’s challenges, opportunities, and problems [1]. Due to overwhelming feelings of helplessness, fear, or anger and the concomitant physiological sensations, self-regulation strategies can fail. Methods like self-talk, which can usually help to re-focus during a demanding situation, may not be initiated. An everyday example for such a demanding situation is the preparation for a test at school by children after blaming themselves for being inadequate or stupid. These students may then end up in a negative spiral after using inadequate coping strategies (e.g., mental or behavioral disengagement), which cause them to experience stress and increase the probability of them abandoning the situation altogether [2]. This adverse outcome is because how students think about their own abilities may influence their emotions as well as the intensity of these emotions and eventually, the likelihood of success at self-direction [3].

With regard to possibilities of stress reduction, eye movement desensitization and reprocessing (EMDR) has been established as an efficient therapeutic intervention concerning posttraumatic stress disorder (PTSD). Evidence also suggests that problems caused by childhood trauma can also be effectively treated by EMDR, both in adults and children [4]. EMDR has also been shown to successfully treat symptoms of anxiety and depression [5] along with subjective distress in PTSD cases [6]. Therefore, while originally developed to ease the burden of traumatic memories on PTSD patients, EMDR is now used to treat a range of ailments that emanate from stressful life experiences [7].

EMDR, which was first described and developed by Shapiro [8], is practiced in eight phases. In its crucial desensitization phase, which follows initial phases such as developing a treatment plan and building up a therapeutic relationship, the subject’s visual focus is directed bilaterally after the subject focuses on a traumatic event and its accompanying physical manifestation [9]. While holding the image, thoughts, or sensations associated with the trauma in mind, brief sets of quick bilateral stimulations are prompted until the disturbances and the dysfunctional cognitions related to the trauma improve [10]. This can increase the subject’s ability to stay present and incorporate a greater sense of self-mastery [7]. While the bilateral stimulations are executed quickly during the desensitization phase as stated above, it is executed slowly in the subsequent installation-phase. The client’s focus is directed on a positive self-assessment towards the situation until the traumatic situation is no longer associated with emotional distress and the now changed perception and feeling during the memory of the event is anchored [7].

The bilateral stimulation of the subject’s focus typically involves the therapist moving a finger from side to side, which the subject follows with his or her eyes. Other forms of stimulation are administered through some signals on the subject’s body, for example, a tap on the left and right shoulders or knees or through sound, for example, alternating aural stimulations from the left and the right side of the subject [11]. These alternating stimulations are seen as the reason for the decreasing intensity of the subjects’ symptoms through EMDR [12] and seem to allow subjects to report positive changes concerning affective, cognitive, and imagery parts of the target on which they focus during an EMDR-session [13]. However, the specific role of the consistent eye movements based on the stimulations in EMDR execution remains uncertain. Eye movements can, therefore, at least be seen as an additional factor in alleviating negative symptoms, instead of only exposure-based treatments [14]. Alterations to the retention of memories that have a negative impact on one’s everyday life appear to work through amygdala deactivation with the help of interventions such as EMDR or working-memory tasks [15]. Bisson et al. [16] view extinction learning as the essence of effective treatments of fear and anxiety in general. In a review of EMDR and its underlying mechanisms, Landin-Romero et al. [10] found neither a uniform indication of which mechanisms play which role in EMDR nor an explanation of how these mechanisms can be precisely measured. Although a scientific integrative model could remedy this, they argue that a comprehensive conceptual basis should be created first.

In a standard EMDR therapy set, 6 to 12 sessions are typical; however, fewer sessions may be sufficient for some people as well [17]. Finally, the type as well as the severity of a trauma determines the number of sessions and the length of the therapy sessions [18]. Low dropout rates in EMDR therapy suggests that the reaction to unpleasant thoughts, pictures, and feelings, i.e., adverse emotions, is well tolerated by subjects [5].

Although EMDR has been found to be helpful and relevant in psychiatric contexts, its partial use may also be investigated in non-clinical environments. Concerning test anxiety, EMDR may be an effective intervention to overcome stress and angst, but so far, findings have been inconclusive. Maxfield and Melnyk [19] found that test anxiety can be reduced by a single 90-min session of EMDR, although the authors indicated the small sample size (*n* = 44) as a limitation of their study. In another non-therapeutic context, e.g., coaching of employees, promising results in applying EMDR (4 participants; 1–10 h) were observed with restored or enhanced work performance and a desensitization towards a disturbing event that had impaired employee performance at work [20]. In a small group of amateur golfers (*n* = 4), Falls et al. [21] found that the use of three EMDR sessions delivered notable changes in detrimental prospective imagery as well as reduction of cognitive and somatic anxiety. Brooker [22] found that both EMDR (therapy group 1) and cognitive hypnotherapy (therapy group 2) decreased anxiety in music performers (i.e., state anxiety) and increased performance confidence in advanced pianists compared to a control group. Additionally, trait anxiety decreased significantly only in the EMDR group. EMDR has also been used to support cognitive and emotional remediation of undergraduate future schoolteachers in their first year of the master program. Students of the EMDR group, unlike those in the control group, experienced a significant decline in their negative emotions as well as an increased sense of their self-efficacy ([23]).

Isolated emotional blockades are often the reason non-clinical subjects look for therapists or coaches who offer para-therapeutic aids. The wingwave^®^ method [24] is a non-therapeutical coaching method that uses the eye movement component of EMDR and combines it with a muscular test named the Bi-Digital O-Ring Test (BDORT), which was originally developed by Omura [25]. The method relies on the subjects forming a “ring” with their index finger and their thumb and the administrator of the method (e.g., mental health professional, coach) trying to open this “ring” with both hands. The clients’ state of health or his/her tolerance of certain medications is then subjectively evaluated by the professional depending on the force that the client is able to apply while holding together the formed ring. Combinations of BDORT and eye movement initially were used in dental studies (e.g., [26,27]). BDORT was used to identify the exact dosage of sedatives used for apprehensive patients prior to dental treatment and which sedative would work best on a patient [26]. BDORT was also used to explicitly avoid possible side effects of medication prior to dental treatment [27]. Although eye movement was a part of those initial studies, additional use of acupuncture as well as parallel unsystematic combination of BDORT and eye movement made it difficult to evaluate this approach and it was therefore more likely to be classified as eclectic.

In the wingwave^®^ method, the muscular strength that the coach measures with the BDORT is not used to evaluate the state of health. It is, however, used to identify the themes in the coaching-process. Since it is evidenced that negative emotional conditions, such as anxiety, lead to a decrease in the strength of the finger musculature [24,28], the wingwave^®^ method makes use of this functionality. As coaches of the wingwave^®^ method are trained to work with this technique, it helps them to identify if the subject is under emotional stress. The easiest evaluation means that the coach would need to utilize less strength to open the subject’s “ring” when the subject is experiencing negative emotions, in contrast to neutral or positive emotions.

Enhanced performance in some sport disciplines have been found to be the outcome of wingwave^®^ coaching. In a pilot study by Rathschlag and Memmert [29], anxiety reduction, both trait and state anxiety, was observed after applying the wingwave^®^ method. In this study, the authors have used a self-developed machine that hydraulically generates a pulling force to separate the thumb and the index finger of the observed subject. The subject’s task was to hold the two fingers together and to form a ring with them and the machine was used to identify the strength of the finger musculature before and after the wingwave^®^ sessions. The authors also collected data on subjectively perceived anxiety on a 9-point Likert scale (LS), which decreased in the course of a 1–2 h intervention with the wingwave^®^ method. All results were compared to the outcomes of a control group, whose participants did not receive any intervention. However, the authors only focused on a timeframe of four weeks between measurements one and two. Furthermore, they focused on subjective measures of state and trait anxiety. Koetter and Niebuhr [30] showed that the general health and state of depression and anxiety deteriorated in medical students while preparing for an examination. The authors also observed that perceived medical school stress could be reduced by short-term resource-oriented coaching sessions. The wingwave^®^ method was partly used in the intervention group, but effects of this intervention could not be evaluated. Naumenko et al. [31] investigated the effects of wingwave^®^ coaching on a mild fear of flying and found promising results after applying two coaching sessions to the clients. However, since there were only four clients, this result should be viewed with caution.

To address the limitations of the studies described above, more objective measures, a longer observation time frame and a bigger sample size for the assessment were taken into consideration in the current study. The most important aim, however, was to reduce not only test anxiety but also address fear and dislike of school. Another aim was investigating the impact of an intervention on concentration performance and subjective feelings towards school subjects.

School anxiety, usually characterized by performance or test anxiety, is a serious issue among schoolchildren today. Test anxiety in particular is characterized by a reaction of the affected student in the form of worry and emotionality in test situations [32]. Students, who suffer from a strong test anxiety, generally tend to avoid stimuli that cause fear, e.g., tests. This can then have an impact on dislike of school or even refusal to attend [33]. Although parents and teachers often realize that a child may be suffering from school-related anxiety, many times, they lack appropriate measures to offer help to the affected children. Overwhelming emotional stress may also negatively impact school performance like oral participation, test performance, oral presentations in front of a class or jeopardize promotion to the next class. In case a transition does not work out for a child, several negative consequences, which can cause acute mental stress, need to be considered. Besides the personal disappointment of not having passed an important test phase, other negative outcomes may occur in the social context (such as classmates continue attending the same school, but the affected child has to leave the established school community). The severity of the personal emotional decline in the child in such situations should not be underestimated. Yamamoto [34] was able to show that children from grades 4 to 6 tend to rate the extent of everyday problems—including those of everyday school life—similar to that of critical life events. In Germany, grade 6 plays a crucial role in determining whether a student is able transition to the next grade and/or is allowed to stay in the same school. This school year can be seen as a particularly delicate and stressful time and is, therefore, chosen as the focus of the current study.

Nitkowski et al. [35] examined the German self-report questionnaire of anxiety (AFS; [36]) and found that between 1974 and 2016, mean scores of the scales of self-report (i.e., dislike of school, manifested anxiety, test anxiety, and social desirability) either did not change or changed only to a negligible extent. Thus, it does not appear that there is a modern type of schoolchild who suffers more from school anxiety than earlier generations of pupils. However, it makes sense to take a closer look at the stressors that schoolchildren often face nowadays. In the next step, it is also important to discuss interventions that could potentially provide relief from these stressors.

The primary aim of the current study was two-fold—first, to evaluate a coaching approach and whether it helps schoolchildren to feel more positively in school; and second, to investigate whether this approach has any measurable effects, i.e., objective parameters. In order to advance research on treatment options for school anxiety, it was focused on the state of school anxiety on schoolchildren, their emotions regarding self-chosen subjects and their concentration ability. The current investigation into the effects of a limited number of coaching sessions with the wingwave^®^ method may, therefore, yield information about potentially helpful interventions for reducing stress and negative emotions regarding school, for school-based performance anxiety, and for concentration improvement. We hypothesized that by applying the wingwave^®^ method, (a) concentration performance in pupils of the 6th grade would improve; (b) school anxiety in pupils of the 6th grade would reduce; and (c) subjective feelings towards self-chosen school subjects would improve.

## 2. Materials and Methods

### 2.1. Participants

A statistical power analysis was performed to estimate the necessary sample size (G*Power 3.1.9.7, Düsseldorf, Germany). The power analysis indicated that a sample size of at least 44 participants would result in a power of 0.95 (alpha level = 0.05, f = 0.25). However, due to a high anticipated dropout rate in intervention studies, we decided to request for 1.5 times as many participants as calculated with G*Power.

A total of 66 participants (34 females and 32 males) took part in the study. All participants were schoolchildren attending 6th grade at the time of the observation period. Students were voluntarily involved via announcements in schools and were randomly assigned to either an experimental group (EG) or a control group (CG) by the principal investigator. Finally, due to sick leaves or incomplete compliance with the study requirements, data sets of only 53 participants (28 females, 25 males) aged 11 to 12 years (*M*_age_ = 11.29 years, *SD* = 0.46 years) were analyzed. Of those 53 subjects, 30 were assigned to the EG (15 females, 15 males) and 23 to the CG (13 females, 10 males). Both groups were similar in age (EG: *M*_age_ = 11.28 years, *SD* = 0.45 years; CG: *M*_age_ = 11.32 years, *SD* = 0.48 years). 

The study was carried out in accordance with the Helsinki Declaration of 1975, and written informed consent was obtained from each schoolchild as well as from their legal guardians prior to the experiment. There was no compensation for participation. Approval was obtained from the lead institution’s (German Sport University Cologne) ethics board (approval number 009/2018, approval date 6 February 2018).

### 2.2. Materials

The concentration performance test “KLT-R” by Düker and Lienert [37] is a paper-pencil performance test that investigates concentration performance, that is, the quality and quantity (temporal) of long-term concentration. It consists of nine blocks of 20 separate tasks each which sequentially have to be completed by the schoolchildren. For each block, there is a time limit of 120 s, regardless of the number of edited tasks/quality of concentration; therefore, the completion of the entire test should take 18 min.

The KLT-R can be used in two forms (KLT-R 4-6: for children from grade 4 to grade 6; KLT-R 6-13: for children from grade 6 to grade 13); for this study, the KLT-R 4-6 was used. Both the KLT-R forms exist in two versions to minimize the chances of cheating in the test by copying results from a fellow student. Each student was randomly assigned one of the two versions for the pre-test (T0); thereafter, each student had to take the version of the test they were not originally assigned two weeks later (post-test T1) and then finally complete the version they were first assigned after a couple of weeks to test their retention (retention test T2). The internal consistency (Cronbach’s alpha) is between α = 0.94 and α = 0.97 regarding correct answers and between α = 0.79 and α = 0.93 for wrong answers. The retest reliability after two months is between $r_{tt}$ = 0.86 and $r_{tt}$ = 0.88 [37].

In addition to the KLT-R, a second paper-pencil inventory, the German anxiety questionnaire for students (AFS; [36]) was used to assess the subjects’ feelings towards school. The AFS is an objective instrument containing a total of 50 items on four different scales: dislike of school, manifested anxiety, test anxiety, and social desirability. The questionnaire was validated by correlating the student responses with school grades, teachers’ appraisal estimates, and self-reported test anxiety [35]. The internal consistency (Cronbach’s alpha) of the scales is between α = 0.73 and α = 0.89. The retest reliability after one month is between $r_{tt}$ = 0.71 and $r_{tt}$ = 0.76 and after 2 months between $r_{tt}$ = 0.55 and $r_{tt}$ = 0.71 [36]. Assessors in this study were trained in advance in the correct use of the three assessment measures.

### 2.3. Procedure

Data were collected at three measuring time points in this study, i.e., in a pre-test (T0), a post-test (T1) about two weeks after the pre-test, and a retention test (T2) around six to eight weeks after the post-test. Prior to the pre-test, demographic information and their selection of two school subjects they wanted to improve in was obtained from all the students. At each of the measuring time points, the students had to fill out two inventories (the KLT-R and the AFS). Furthermore, the pupils were required to give a subjective evaluation of their feelings towards the two previously chosen school subjects on a scale from −10 (most negative feeling towards the subject) to +10 (most positive feeling towards the subject).

Between T0 and T1, children of the EG received three wingwave^®^ coaching sessions, each for a duration of one hour. For children of the CG, there was no intervention. The coaching sessions were executed by trained coaches of the wingwave^®^ method who had at least two years of experience in coaching children. Coaches were randomly assigned to the subjects. The coaches did not assess the AFS and the KLT-R measures. As stated above, Rathschlag and Memmert [29] had used a machine to identify the strength of the finger musculature before and after wingwave^®^ sessions. In the present study, the coaches performed the muscular test manually, which is the usual procedure of the wingwave^®^ method and has been applied in a number of previous studies with the wingwave^®^ method [24] and other combinations of BDORT and EMDR (e.g., [26,27]).

For the current study, through a survey prior to the observation, 23 teachers, 105 students of the target group, and experienced wingwave^®^ coaches were asked to state possible reasons for school stress, dislike of school, test anxiety, and lack of concentration in class or at home concerning school-oriented tasks for this age group. From this survey, the 35 most frequently stated causes (i.e., the causes that had been mentioned at least three times throughout the survey) were listed (see Supplementary Materials), which was then handed out to the coaches, prior to their coaching sessions with the students. If the students did not mention any stress words themselves at the beginning of the coaching, the words from the list were tested (beginning with the first word on the list) by means of the wingwave^®^ method.

The coach started the sessions by calling out words or phrases that had been collected and were assumed to cause emotional stress to the students. Shortly thereafter, the coach tested the strength of the “ring” that the subject tried to keep it closed. If the subject succeeded and the “ring” remained closed while the coach tried to pull it open, the coach continued by calling out another word or phrase and testing the subject’s strength once again. As soon as the naming of a word led to a decrease in (muscular) strength (i.e., as soon as the “ring” opened), the respective word became the primary theme of the coaching from then on.

To identify the root cause of the decrease in muscular strength, the coach tried to uncover any painful or emotionally disturbing memories connected to the identified word. A standard manual [24] to guide this process was used by the coaches. Through this manual, all details of the respective memories (e.g., age, setting) were tested until the root cause could be narrowed down to a singular event or a specific number of events in a defined time frame. As soon as the root cause was uncovered, the subject was asked to identify the bodily sensation (e.g., coldness, warmth, tingling sensation, belly rumble) concurrent with the experienced emotion connected to that particular memory. In some cases, no corresponding memory could be revived by this method; however, the subject’s strength declined on mentioning the initial word or phrase. The subsequent procedure was, nevertheless, executed in such cases. 

The coach then used specific finger movements to initiate the subject’s rapid eye movement (REM), a technique that is also used in EMDR. The subjects were asked to focus on their bodies during the implementation of each REM set. The REM exercise was then repeated in sets of 10 to 20 s, until the body sensation disappeared. Once the subject stated that the emotion was no longer tangible or that he/she felt generally good, the coach again tested the previously identified word that was connected to the initial decreased muscular strength. If the subject’s “ring” could now not be opened by the coach anymore, the corresponding theme was marked completed, and process would continue with the next word. If, on the other hand, the “ring” could still be opened by the coach, an underlying second influence, and hence, a second root cause for the strength decrease was identified and approached just as the first root cause, as stated above. 

In total, five coaching topics (e.g., tension during a math test, boredom during French lessons) were identified for each subject. The first two of the three coaching sessions focused on these five topics. In the third coaching session, positive emotions and beliefs (i.e., resources) that may support the subject, were identified. At first, the focus was on the ability to concentrate. Here, the aim was to link the state of increased concentration to a test or a quiet working phase in school. The coach first asked the subjects to visualize a situation in which they wanted to feel more focused in the future. Then, they were asked to recall a moment in the past in which they felt concentrated and to focus on their accompanying body sensations. Shortly after that, the coach used specific finger movements to initiate the subject’s slow eye movement. This slow eye movement is assumed to intensify and strengthen the potentially helpful emotion and to link this modified state to the previously visualized scenario (cf. the installation-phase in the EMDR protocol [7]). Thus, in the next comparable situation, the subject is supposed to feel powerful and well-equipped to face the stimulus that was previously perceived as negative [24].

After that, the coaches called out phrases that were suspected to be the underlying problematic self-perception (e.g., “I can’t concentrate”). These phrases had been summarized and transformed to the school setting from a list of beliefs in the initial wingwave^®^ literature [24]. The words from the list were subsequently tested with the muscular test. Every weak test outcome resulted in a sharper focus on the underlying topic in the same manner as mentioned above. This way, the focus of the coaching was on both, typical situations and settings in school, as well as general personal beliefs.

## 3. Results

All data were analyzed with IBM SPSS Statistics (Version 26). A 2 × 3 (group [EG, CG] × time of measurement [T0, T1, T2]) multivariate analysis of variance (MANOVA), with repeated measures on the last factor and the responses/scores for the different subscales as dependent variable, was conducted. The MANOVA was performed to examine the relative differences in performance of the subscales (KLT-R total answers, KLT-R correct answers, AFS test anxiety, AFS manifested anxiety, AFS dislike of school, AFS social desirability, subjective feeling) (A z-transformation for all data was calculated. To ensure better articulation, however, the results are reported in their original form, as the nominal values can, therefore, be seen more clearly in the context of the selected questionnaires and test). To understand and analyze any significant effects in further detail, we performed separate post hoc comparisons. Bonferroni-corrected pairwise comparisons were used to follow up significant primary effects. The results of these analyses are shown in different figures. For analyses in which the sphericity assumption was violated, we reported the value of ε from the Greenhouse-Geisser correction.

### 3.1. Multivariate Results

Using Pillai’s trace, there was no significant effect of group on the dependent variables—KLT-R total answers, KLT-R correct answers, AFS test anxiety, AFS manifested anxiety, AFS dislike of school, AFS social desirability, subjective feeling, *V* = 0.17, *F*(1.27, 45), *p* = 0.29. There was a significant effect of time on all dependent variables, *V* = 0.77, *F*(9.31, 38), *p* < 0.001, as well as a significant effect of time and group, *V* = 0.62, *F*(4.45, 38), *p* < 0.001.

When comparing the baseline level of the variables, it was noticeable that the groups only differed with regard to subjective feeling, *t*(51) = 2.84, *p* = 0.042. For all other variables, there were no significant differences (all *p*s > 0.185; see Table S1 in Supplementary Materials). The results were assessed using six independent Bonferroni-corrected t-tests. However, this is not an indication that the interaction effect is due to these initial differences since the experimental group had a significantly lower level of subjective positive feeling than the control group, which eventually increased to a level that was higher than the level of the control group at both post-test and retention test times.

In order to test the hypotheses stated above, the main effects as well as the effects of the interaction between time and group on the variables have been reported in detail in Table 1. In Table 2, mean and standard variation of all relevant variables at the three measurement times are presented.

### 3.2. Post Hoc Analyses

**Figure 1:**
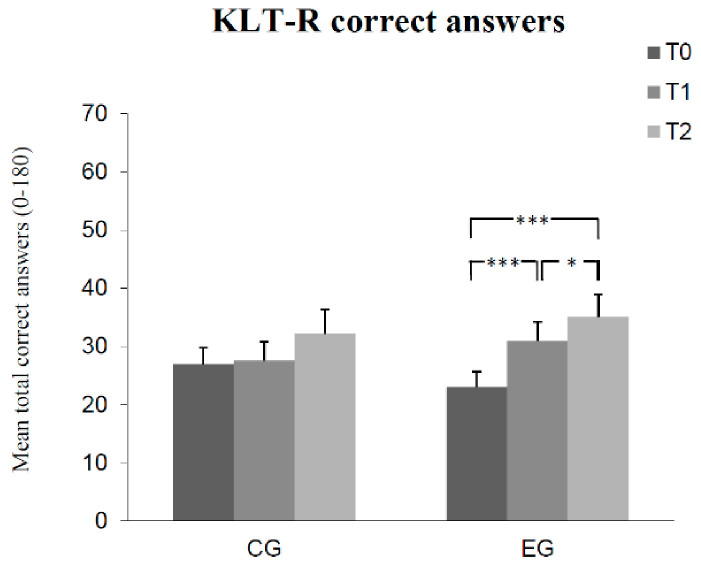
Mean differences (and SE) in absolute correct answers among groups (control group CG, experimental group EG) as a function of measurement time (T0, T1, T2). (Effect sizes Cohen’s d: T0 to T1 = 0.48, T1 to T2 = 0.22, and T0 to T2 = 0.67 in the EG). (Notes: *** *p* < 0.001, * *p* < 0.05. All *p*-values are Bonferroni-corrected.)

**Figure 2:**
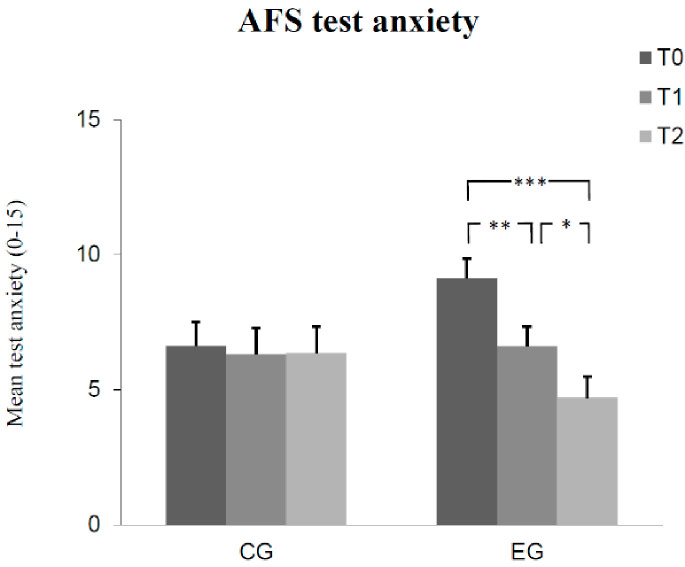
Mean differences (and SE) in test anxiety among groups (control group CG, experimental group EG) as a function of measurement time (T0, T1, T2). (Effect sizes Cohen´s d: T0 to T1 = 0.63, T1 to T2 = 0.46, and T0 to T2 = 1.07 in the EG). (Notes: *** *p* < 0.001, ** *p* < 0.01, * *p* < 0.05. All *p*-values are Bonferroni-corrected.)

**Figure 3:**
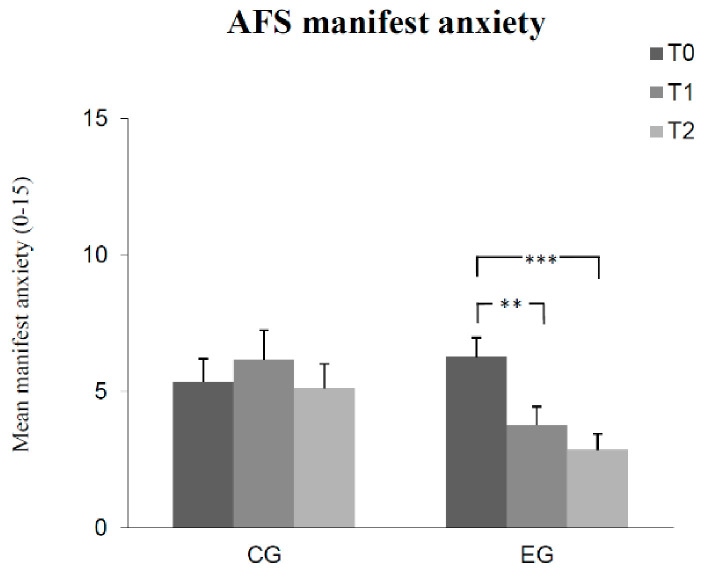
Mean differences (and SE) in manifested anxiety among groups (control group CG, experimental group EG) as a function of measurement time (T0, T1, T2). (Effect sizes Cohen´s d: T0 to T1 = 0.67 and T0 to T2 = 0.96 in the EG). (Notes: *** *p* < 0.001, ** *p* < 0.01. All *p*-values are Bonferroni-corrected.)

**Figure 4:**
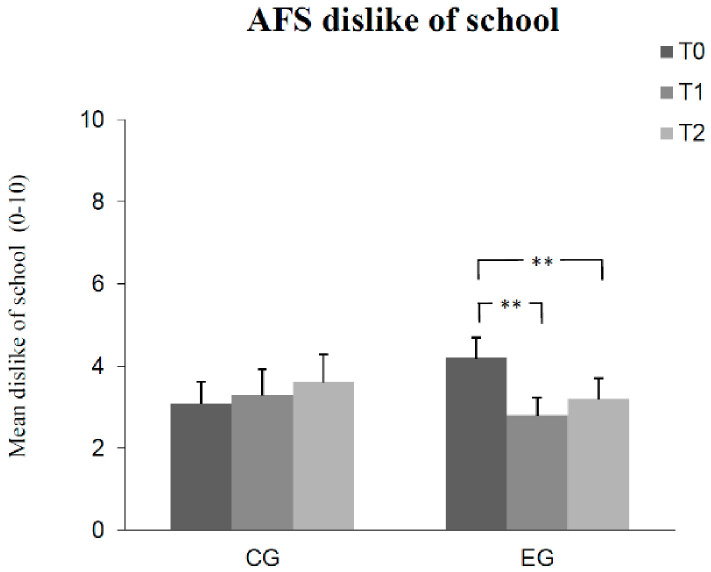
Mean differences (and SE) in dislike of school among groups (control group CG, experimental group EG) as a function of measurement time (T0, T1, T2). (Effect sizes Cohen´s d: T0 to T1 = 0.55 and T0 to T2 = 0.37 in the EG). (Notes: ** *p* < 0.01. All *p*-values are Bonferroni-corrected).

**Figure 5:**
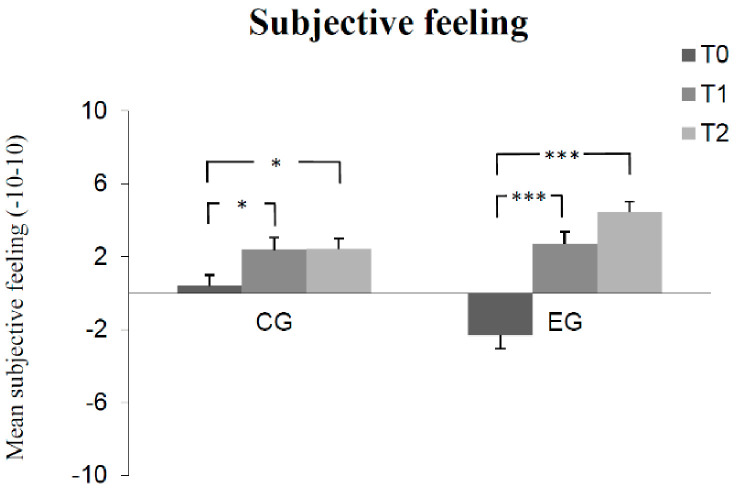
Mean differences (and SE) in subjective feeling among groups (control group CG, experimental group EG) as a function of measurement time (T0, T1, T2). (Effect sizes Cohen´s d: T0 to T1 = 0.58 and T0 to T2 = 0.63 in the CG; T0 to T1 = 1.35 and T0 to T2 = 1.84 in the EG). (Notes: *** *p* < 0.001, * *p* < 0.05. All *p*-values are Bonferroni-corrected).

The results of Bonferroni-adjusted post hoc analyses can be seen in Figure 1, Figure 2, Figure 3, Figure 4 and Figure 5. The number of correct answers increased significantly from pre-test to post-test, as well as from post-test to retention test in the experimental group, while no significant change of correct answers was measured in the control group at each of the measured times. Test anxiety decreased significantly from pre-test to post-test, as well as from post-test to retention test in the experimental group, while no significant change of test anxiety was measured in the control group at each of the three measurement times. Manifested anxiety as well as dislike of school decreased significantly from pre-test to post-test, as well as from pre-test to retention test in the experimental group, while no significant change of manifested anxiety and dislike of school was measured in the control group at any time. Subjective feeling increased significantly from pre-test to post-test, as well as from pre-test to retention test in both the experimental group and the control group.

## 4. Discussion

In this study, the application of the wingwave^®^ method appeared to be successful in reducing the individual emotional distress among the subjects comprising German students of grade 6. Following Rathschlag and Memmert [29], who had evaluated the wingwave^®^ method in a more global context, the authors in this study focused on school anxiety (measured via the AFS) and concentration performance (measured via the KLT-R). Previous findings have shown that, on many occasions, past events that were associated with negative emotional experiences—so-called performance stress imprinting (PSI; [24])—were somehow linked to present elements of school life (e.g., tests, homework, school subjects per se, or teachers). As soon as those present elements were mentioned in the coaching sessions of the current study, weaker muscular activity in the finger test was observed, indicating emotional stress (e.g., anxiety, helplessness, sadness). Identifying the root cause of the negative emotions and focusing on present initial emotional stimulation and associated physiological symptoms followed by intervention through the guided movement of the eyes (i.e., the desensitization phase) led to a decrease in perceived stress. Significant positive changes in test anxiety, manifested anxiety, and dislike of school indicate that three sessions with the wingwave^®^ method lead to improvements in the emotional state about school in general and different aspects of school in our study subjects pool. Additionally, concentration performance improved during the process of the three coaching sessions. This may be seen as an independent effect through focus on the concentration ability of the schoolchildren, especially in the last coaching session and as an effect of the decreased school anxiety—which possibly began with the first coaching session itself. Lastly, the subjective feeling towards the two self-chosen subjects of the schoolchildren in the experimental group increased from pre-test to post-test as well as from pre-test to retention test. Considering that the experimental group initially had a significantly lower level of subjective positive feeling than the control group, this is even more remarkable. Thus, the thoughts and feelings towards school among students who underwent three coaching sessions with the wingwave^®^ method changed to a level in which school anxiety could decrease while the ability to concentrate could increase. This is not only remarkable with regard to the noticeable effect, but also for the duration for which this change lasts. The feelings of positivity and the consequent changes in the anxiety and concentration levels did not last only for two weeks, but remained stable (AFS manifested anxiety; AFS dislike of school) or even increased for six to eight weeks after the final coaching session without any additional intervention on two of the measured parameters (KLT-R correct answers; AFS test anxiety). Finally, the low dropout rate makes attrition bias due to incomplete outcome data unlikely.

Beyond the results of Foster and Lendl [20], it could be shown that only three coaching sessions can have a positive effect and an enhanced in a school context may be observed. In contrast to Koetter and Niebuhr [30], it was observed that the three coaching sessions with the wingwave^®^ method can have a significant effect without any pure psychoeducational intervention. As opposed to the EMDR intervention on students by Maxfield and Melnyk [19], the current study found that school anxiety might be reduced by means of the wingwave^®^ method. In addition to the influence of an alternating study design, this deviation could also be due to the fact that the muscular test is included in the wingwave^®^ method. This concomitant tool could have been useful as a supplementary feedback for students and coaches during the interventions. The students could not hold their fingers together when coaches assessed the impact of the stress words or when students remembered stressful memories. However, they could do that after successful bilateral stimulation. This—with regard to EMDR—additional tool may have anchored the change and led to feelings of positivity. The variety of coaches participating in this study can also be seen as an interesting approach in comparison to studies with limitations due to few professionals conducting EMDR interventions (e.g., [38]). Lastly and most importantly, the systematic combination using a protocol rather than an eclectic combination of BDORT and bilateral stimulation [26,27] can be seen as a promising approach to widen the horizons of pragmatic and effective use of bilateral stimulation in a non-clinical context.

There are some limitations of the current study and considerations for future research that should be addressed. As a first study limitation, the control group did not undergo any intervention, and therefore, this makes it impossible to rule out non-specific effects of a treatment. In the past, researchers compared trauma-focused cognitive-behavioral therapy and EMDR over their impact on children and adolescents for posttraumatic stress symptoms; cf. [39]. In our study, an alternative established intervention could have added more value. In addition, the sole focus on certain issues through the use of the muscular test could consequently have become the dominant factor, rather than a part of the EMDR intervention. It remains unclear whether the stress-reducing effectiveness of the coaching sessions employed in this study is rooted in specific interventions or the combination of all methodological components or whether it is merely an unspecific effect of support. Whether the decreased anxiety during test taking (coaching session one and two) or the increased use of individual resources that the third coaching session focused on led to increased concentration performance remains unclear. This generally applies in this study since the program of the three coaching sessions contained deficit-oriented and resource-oriented approaches and the effect of each effect individually was not surveyed. The assessment of subjective feeling left the authors of this study with ambiguous results. Although the experimental group showed lower numbers in the pre-test and higher numbers in the retention test than the control group, the numbers grew in both groups throughout the measurement periods. Both groups had started with significantly different baseline values on subjective feeling. It may, therefore, be that the subjective feeling of the experimental group was possibly at the lower end of the baseline, and so, the increase throughout the process should be interpreted with caution. Although, in this study, more participants took part than in studies with a comparable approach (e.g., [22,23]), larger sample sizes should be considered for future studies in order to increase the validity of the data. Lastly, objective physiological measures (e.g., heart rate variability or electromyogram) could also have increased the validity of this study and should be considered in future studies with the wingwave^®^ method when anxiety and/or concentration parameters are collected. Additionally, the use of a machine to identify the strength of the finger musculature (cf. study design of [29]) could have added more objectivity to the study. For this reason, the contributions made by the individual method components remain indistinguishable. As a counterargument, the wingwave^®^ method is carried out through a standardized procedure, exactly the same way, manually by each coach to identify the subject’s finger strength. This is an integral part of the basic wingwave^®^ protocol.

It could not discernibly be guaranteed that all the children reported their memories accurately—intentionally or unintentionally. However, based on the follow-up sessions and the experiences of the coaches in the test settings, we assume that none of the participants intentionally reported any false memories. This issue of unintentional or intentional possible false memories should be considered while designing future studies.

The coaches in this study had been working exclusively or predominantly with children prior to the experiment and were briefed about the context of this study. They were selected based on their competence in dealing with strong emotions during coaching for children. The students and parents had the contact details of the coaches assigned to them just in case they needed help between coaching sessions, though none of the students made use of this. For the retention test, the authors saw all the students six to eight weeks after their final coaching session and followed up on the outcomes of the experiment. All the children reported that they were satisfied with both the coaching sessions and the coaches they were matched with. The coach-student pairing as a potentially disruptive factor and an opportunity for low threshold communication for children and parents should be focused on in future studies as these were cited as important by both the children and the parents in this study.

It may be worth making quick and reliable professional mental health available in situations where the coach might be feeling overstrained by the severity of the burden on clients during the reporting of potential traumatic memories. To additionally counter this risk, in addition to assessing anxiety parameters, psychiatric disorders can also be evaluated in future studies prior to the start of such study to exclude subjects for whom the wingwave^®^ method could possibly pose a risk during the course of the experiment.

The total timeframe of eight to ten weeks employed in the current study offers supporting evidence for the conclusion that a degree of time stability (i.e., 6–8 weeks) of the effects of the coaching sessions was reached. Future studies implementing retention tests after several months could re-validate this effect. An additional psychoeducational input, as Koetter and Niebuhr [30] included in their approach, could also add to the effects of an intervention in this context. Even a marginal utility of such an approach is to be considered in future research projects. In addition to the added value, this is due to the advantages of using psychoeducation in groups of two or more subjects. Due to simultaneous care of more than one person with psychoeducational measures, this approach can be seen as promising for reasons of time efficiency compared to 1 to 1 care in a traditional coaching setting.

## 5. Conclusions

The findings of this study may help teachers and children in a classroom environment in the future. As De Corte et al. [2] pointed out, teachers and other specialists in classrooms (e.g., psychologists or social workers) can create a performance-friendly and benevolent environment and teach schoolchildren the skills to cope with and regulate their emotions on their own. These specialists should also deepen their knowledge and acquire appropriate tools and methods to fulfill this task. If further studies confirm that the wingwave^®^ method can, in fact, alleviate anxiety in schoolchildren and increase concentration performance, it could be a recommended intervention. This is particularly useful if the positive effects not only have a short- or medium-term effect as described here, but also lead to long-term benefits that can have a functional utility in a variety of domains. If performances in school (e.g., grades) would be assessed parallel to coaching interventions, the subjective benefit at an emotional level could possibly be supplemented by teachers rewarding the schoolchildren. Furthermore, focusing on preventive psychoeducation in school offers the perspective of organizational and financial advantages compared to individual coaching. Stress in students, especially due to upcoming exams, could be focused on in future studies, as the wingwave^®^ method addresses individual biographical stressors as well as the stress attributable to the exam situation.

In summary, the wingwave^®^ method appears to be an effective choice for short-term coaching agendas and can potentially be used in various fields of application. Future research will have to specify its effectiveness and further investigate the effects of wingwave^®^ coaching in comparison to other evidence-based interventions.

## Figures and Tables

**Table 1:** MANOVA results representing the main effects of group, time, and the interaction between group and time on the variables KLT-R total answers, KLT-R correct answers, AFS test anxiety, AFS manifested anxiety, AFS dislike of school and Subjective feeling (Note: In case of violation of the sphericity assumption, Greenhouse–Geisser ε is reported).

	Effect	F	df	p	η^2^	ε
KLT-R total answers	group	0.35	1, 51	0.556	-	-
	time	16.47	1.63, 83.09	<0.001	0.24	0.82
	group * time	1.15	1.63, 83.09	0.310	-	0.82
KLT-R correct answers	group	0.03	1, 51	0.871	-	-
	time	17.15	1.49, 76.10	<0.001	0.25	0.75
	group * time	3.72	1.49, 76.10	0.041	0.07	0.75
AFS test anxiety	group	0.13	1, 51	0.716	-	-
	time	12.98	2, 102	<0.001	0.20	-
	group * time	10.12	2, 102	<0.001	0.17	-
AFS manifested anxiety	group	1.54	1, 51	0.221	-	-
	time	10.27	2, 102	<0.001	0.17	-
	group * time	11.08	2, 102	<0.001	0.18	-
AFS dislike of school	group	0.01	1, 51	0.924	-	-
	time	2.35	2, 102	0.100	-	-
	group * time	5.48	2, 102	0.006	0.10	-
Subjective feeling	group	0.03	1, 51	0.868	-	-
	time	44.99	2, 102	<0.001	0.47	-
	group * time	12.05	2, 102	<0.001	0.19	-

**Table 2:** Mean and standard deviation (SD) of pre-test, post-test, and retention test on the variables KLT-R total answers, KLT-R correct answers, AFS test anxiety, AFS manifested anxiety, AFS dislike of school, and subjective feeling.

	Time of Measurement					
	Pre-Test		Post-Test		Retention Test	
	Mean	SD	Mean	SD	Mean	SD
KLT-R total answers						
Experimental group	32.37	14.78	38.60	18.72	43.43	21.41
Control group	37.78	14.57	41.09	21.16	44.26	21.33
KLT-R correct answers						
Experimental group	23.00	14.83	30.93	18.01	35.13	20.79
Control group	26.96	13.43	27.61	15.36	32.30	19.89
AFS test anxiety						
Experimental group	9.13	3.98	6.60	4.07	4.70	4.28
Control group	6.61	4.25	6.30	4.70	6.35	4.74
AFS manifested anxiety						
Experimental group	6.27	3.86	3.77	3.65	2.87	3.17
Control group	5.35	4.09	6.17	5.16	5.13	4.22
AFS dislike of school						
Experimental group	4.20	2.72	2.80	2.38	3.20	2.75
Control group	3.09	2.52	3.30	2.99	3.61	3.24
Subjective feeling						
Experimental group	−2.32	3.34	2.70	4.08	4.45	4.00
Control group	0.41	3.65	2.39	3.17	2.43	2.75

## Data Availability

The data and test protocols are available on request to the first author.

## Supplementary Materials

The following are available online at https://www.mdpi.com/article/10.3390/children8121102/s1. A list including the German words and Table S1.
