# Supplementary material for: Anxiety Reduction and Improved Concentration in Schoolchildren through Wingwave® Coaching
Source: Children (Basel). 2021 Nov 30;8(12):1102. doi: 10.3390/children8121102 (PMC8700058; doi:10.3390/children8121102)
Supplement: Supplementary file 1 [file children-08-01102-s001.zip › children-1399623-supplementary.pdf]

Supplementary Material

Supplements

List of the 35 most frequently stated causes from the survey.

**Geräusche**	**Sich selbst ablenken (Ohrwurm, bestimmte Gedanken etc.)**	**Stress**	**Nahende Zeugnisse**	**Mitschüler**
**Lautstärke, Lärm**	**Witze**	**Schmerz**	**Im Mittelpunkt stehen**	**Sitznachbar**
**Reingerufe**	**Hausaufgaben (auch Gedanken daran)**	**Schlechte Luft**	**Rolle in der Klasse (z.B. Klassenclown, Tollpatsch)**	**Freund / Freundin**
**Unruhe**	**Klassenarbeit (auch Gedanken daran; „es kommt wieder das dran, was ich nicht gelernt habe")**	**Hohe Erwartungen (der Lehrer, der Eltern etc.)**	**Streber**	**Lehrer**
**Stille**	**Vokabeltest**	**Eltern (getrennt, Stress, Erwartungen)**	**verliebt sein**	**Klasse (z.B. Größe)**
**Lachanfälle**	**Farbe des Stifts der Lehrer bei Korrektur der Arbeit**	**Geschwister**	**Langeweile**	**Tische**
**Ablenkung durch andere**	**Handy**	**Fach (Sport, Englisch, Mathe etc.)**	**Sinnfrage („wofür mache ich das hier?")**	**Material (Unterlagen, Hefte, Stifte etc.)**

Table S1. Means (M), Standard Deviations (SD), t-values (t), degrees of freedom (df) and p-values (p) of Bonferroni corrected t-tests for the baseline-values

	**M**	**SD**	**t**	**df**	**p**
**KLT-R total answers | T=0**					
Experimental group	32.37	14.78	1.33	51	> .99
Control group	37.78	14.57			
**KLT-R correct answers | T=0**					
Experimental group	23.00	14.83	1.00	51	> .99
Control group	26.96	13.43			
**AFS test anxiety | T=0**					
Experimental group	9.13	3.98	-2.22	51	.186
Control group	6.61	4.25			
**AFS manifested anxiety | T=0**					
Experimental group	6.27	3.86	-.84	51	> .99
Control group	5.35	4.09			
**AFS dislike of school | T=0**					
Experimental group	4.20	2.72	-1.52	51	.804
Control group	3.09	2.52			
**Subjective feeling | T=0**					
Experimental group	-2.32	3.34	2.84	51	.042
Control group	.41	3.65			
